# *Caenorhabditis elegans* as a Model System for Duchenne Muscular Dystrophy

**DOI:** 10.3390/ijms22094891

**Published:** 2021-05-05

**Authors:** Rebecca A. Ellwood, Mathew Piasecki, Nathaniel J. Szewczyk

**Affiliations:** 1 Medical Research Council (MRC) Versus Arthritis, Centre for Musculoskeletal Ageing Research, Royal Derby Hospital, University of Nottingham, Derby DE22 3DT, UK; rebecca.ellwood@nottingham.ac.uk (R.A.E.); mathew.piasecki@nottingham.ac.uk (M.P.); 2 National Institute for Health Research, Nottingham Biomedical Research Centre, Derby DE22 3DT, UK; 3 Ohio Musculoskeletal and Neurologic Institute, Ohio University, Athens, OH 45701, USA; 4 Department of Biomedical Sciences, Heritage College of Osteopathic Medicine, Ohio University, Athens, OH 45701, USA

**Keywords:** DMD, *C. elegans*, dystrophin, muscle

## Abstract

The nematode worm *Caenorhabditis elegans* has been used extensively to enhance our understanding of the human neuromuscular disorder Duchenne Muscular Dystrophy (DMD). With new arising clinically relevant models, technologies and treatments, there is a need to reconcile the literature and collate the key findings associated with this model.

## 1. Introduction

Duchenne muscular dystrophy (DMD) is an inherited X-linked recessive disorder characterised by progressive muscle degeneration and weakness, with onset in early childhood [1]. DMD is the most common muscular dystrophy in children, with an incident rate of approximately 1 in 3500 live male births [2]. DMD is caused by mutations in the dystrophin gene, which is the largest known human gene and encodes a set of dystrophin proteins, the major one being a 427 kDa protein. It is primarily located in muscles, but small amounts are also present in neuronal cells [3]. Despite our extensive knowledge of dystrophin and its associated dystrophin glycoprotein complex (DGC), a cure remains elusive.

There are over 60 animal models for DMD that are currently available including the dystrophic *mdx* mouse [4], dystrophin deficient rats [5,6] and the golden retriever DMD model [7]. Although these models are invaluable, they require a long gestation period, a significant amount of space and care, and the following of strict ethical guidelines. A model to study human diseases that bypasses the aforementioned limitations is *Caenorhabditis elegans*. Studying muscular dystrophy in *C. elegans* results in more rapid and more cost-effective experiments; they are simple to feed and maintain throughout their short lifespan (approximately 3 weeks). The *C. elegans* DMD model was first reported by Segalat and colleagues in 1998 and has since been applied in a number of disease focused studies [8,9]. A comparison of some of the different animal models that have been used to study DMD can be found in Table 1.

In *C. elegans,* the homologue for mammalian dystrophin is *dys-1*, which encodes for a dystrophin-like protein called DYS-1. In *C. elegans* there are two known isoforms of DYS-1: DYS-1A and DYS-1B. Whilst DYS-1A is almost analogous to human dystrophin, DYS-1B only corresponds to the last 237 amino acids of isoform A [15]. DYS-1 has been shown to be expressed in the body wall, head, pharyngeal and vulva muscles [20].

**Figure 1:**
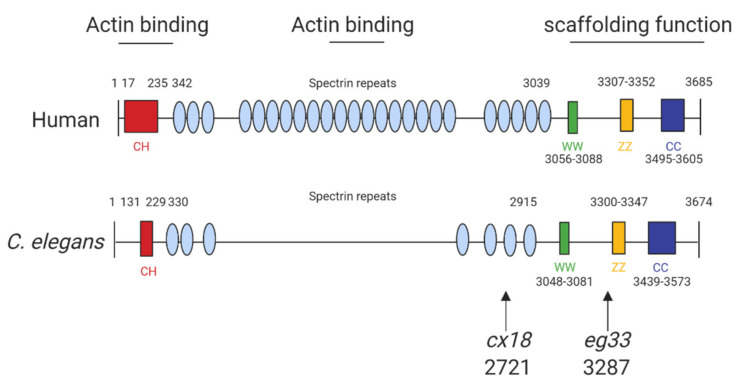
Structure of human and *C. elegans* dystrophin proteins. The structures of human dystrophin and *C. elegans* DYS-1. The size of human and *C. elegans* dystrophin is almost equivalent. They also share similarities in key motifs: CC, coiled coil domain; CH, calponin homology domain (“actin-binding” domain); WW, domain with two conserved W residue; ZZ, zinc finger domain. The arrows indicate the amino acid positions of the mutation sites for the commonly used mutants: *cx18* and *eg33* alleles, which are both nonsense mutations. Adapted from Oh and Kim (2013) [13] and Gieseler et al. (2017) [15]. Created with biorender.com.

The structure of human dystrophin and *C. elegans* share extensive sequence similarities (Figure 1) and introducing mutations into *C. elegans dys-1* gives clinically relevant phenotypes (Table 2). The main models used are *dys-1(cx18), dys-1(cx18;hlh-1)* and *dys-1(eg33)*, and the position of these mutations can be seen in Figure 1. To further support the usefulness of this model, the introduction of human dystrophin cDNA is able to rescue these phenotypes [13].

**Figure 2:**
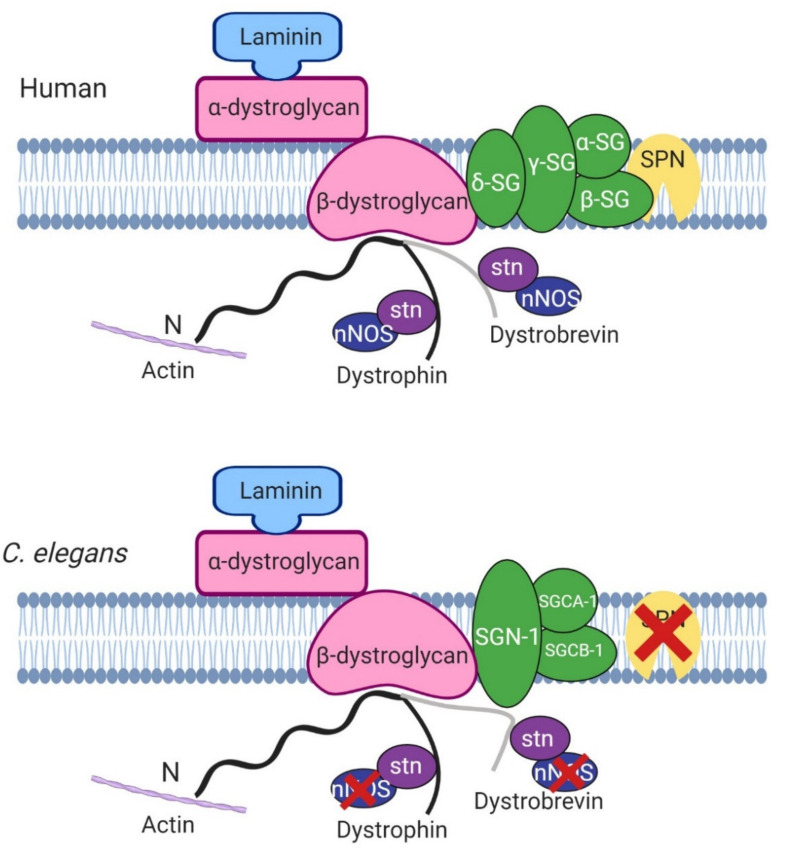
Basic structure of human and *C. elegans* dystrophin glycoprotein complex. Most of the proteins in the mammalian model are found in *C. elegans* apart from sarcospan (SPN) and nitric oxide synthase (nNOS). SG, sarcoglycans, stn, syntrophin. Adapted from Grisoni et al. (2002) [10]. Created with biorender.com.

One of the roles of dystrophin is to link the cytoskeleton, the sarcolemma and the extracellular matrix (ECM) by binding cortical F-actin via its N-terminus and DGC proteins via its C-terminus. The DGC is a monomeric complex that is composed of at least 10 different proteins including dystroglycans, sarcoglycans, sarcospan, dystrobrevins and syntrophin (reviewed by Blake et al. 2015 [3]). There is significant evidence of conserved homologues for the other components of the DGC in *C. elegans*, further highlighting the usefulness of *C. elegans* in the study of DMD (Figure 2).

Despite the proven usefulness of the *C. elegans* model, it also has its limitations. *C. elegans* have a very simple body plan, they have similar protein networks to humans (but the exact nature of the interactions is different due to the varying levels of homology between genes), they lack satellite cells and lack a conventional inflammatory system [15,29]. Nevertheless, this model has been extensively utilised and has provided us with useful insights into this complex disease. As there has not been a dedicated review in almost two decades [30,31], there is a need to reconcile the literature and collate the key findings associated with this model.

## 2. Phenotypes Observed in *C. elegans* DMD Mutants

### 2.1. The dys-1 Single Mutant

The dystrophin homologue in *C. elegans*, *dys-1*, was identified over 20 years ago. Loss-of-function mutations of the *dys-1* gene (*dys-1(cx18/cx26/cx35/cx40/ad538*)) made animals hyperactive and slightly hypercontracted. Additionally, the *dys-1* mutants were found to be hypersensitive to acetylcholine and to the acetylcholinesterase inhibitor aldicarb, suggesting that *dys-1* mutations affect cholinergic transmission. Interestingly, these mutants appeared to have normal muscle cells. Moving forwards the worm mutant *dys-1(cx18)*, which has a nonsense mutation at amino acid 2721 (Figure 1), was predominantly used as its phenotypes could not be distinguished from carboxy-terminal deletions of the gene [20].

### 2.2. Enhancing the Phenotype of dys-1 Mutants

In patients with DMD, severe muscle degeneration is a well-recognised phenotype. As mutations in *dys-1* did not result in muscle degeneration as would be expected, it was hypothesised that this was likely due to the short lifespan of the animals, as this phenotype can take a long time to become present in mammals [20]. The *mdx* mouse model, akin to the *dys-1* single mutant, has a mild phenotype and thus is not the best model for human DMD. To improve the mouse model, double knockout mice were generated lacking dystrophin and MyoD which display severe muscle degeneration [32], and it was thought combining a mutation in *hlh-1* (*C. elegans* homologue of MyoD) with *dys-1* would give a time-dependent muscle degeneration that was lacking in the original single mutant. This led to the generation of the *dys-1(cx18);hlh-1(cc561ts)* mutant [24]. This double mutant had a similar phenotype to its predecessor but in addition it had severe muscle degeneration and an egg laying defect [24]. This double mutant has been used widely; however, the lack of understanding of the mechanism of these enhanced muscular degeneration effects may impact upon its translational importance.

### 2.3. Novel Mutation in dys-1

More recently, a newer mutation has been discovered which has a nonsense mutation at position 3287 (*dys-1(eg33*)). It is apparent that this model may be the most clinically relevant, as, unlike prior models carrying mutations in *dys-1*, this mutant shows muscle degeneration without the need for a sensitised background [13]. Furthermore, it has been shown to have similar locomotory defects to the previous two models but with increased severity. Various aspects of the *dys-1(eg33)* swimming environment have been assessed using the *C. elegans* Swim Test (CeleST), and these animals were deficient in almost all swimming measures [12,26]. To support that *dys-1(eg33)* is a more clinically relevant model, a recent study compared *dys-1(eg33)* and *dys-1(cx18)* [11]. *dys-1(eg33)* was found to be weaker, to exhibit a more severe decline in locomotion and have severe mitochondrial fragmentation compared to *dys-1(cx18)* and wild-type (WT) animals [11,12]. These animals were also shown recently to have altered pharyngeal pumping demonstrating a loss of calcium homeostasis [12]. Pharyngeal pumping has been proposed as a potential model for the heart in *C. elegans* as both are made of striated muscle, contract rhythmically and both are controlled by evolutionarily conserved genes [33]. However, it remains unclear how useful a model worm pharyngeal muscle is for cardiac dysfunction but a decline in pumping in the DMD model raises interesting possibilities [12]. Table 2 shows all known phenotypes associated with each of the models discussed.

## 3. Effects of *dys-1* on Gene Expression

The first gene expression profiling study was conducted by Towers et al. (2006) [34] on *dys-1(cx18)* and *dys-1(cx35)* compared to WT using DNA microarray technology [34]. Gene expression profiling identified 44 upregulated and 71 downregulated probe sets. Of these genes, only 10 had human orthologues but the categories of regulated genes were akin to those from DMD patients. The most prominent of upregulated genes were “cell surface and extracellular matrix”, and collagen genes especially were highly upregulated. This is consistent with patients as damaged muscle cells are gradually replaced by collagen-rich, fibrous tissue [34,35]. For downregulated genes it was “intracellular signalling and cell–cell communication” (particularly neuropeptide-like proteins), this is inconsistent with human muscle biopsies where “energy metabolism and mitochondria function” was the largest represented group [34]. This discrepancy may be explained by the fact that in humans, muscles go through a cycle of degeneration and regeneration which requires energy [36]. As *C. elegans* lack satellite cells they are unable to regenerate muscle [37]. Other groups that were similar in *C. elegans* and in patients were the upregulation of the immune response, downregulation of development and growth genes and the downregulation of muscle proteins (particularly UNC-89 in *C. elegans*) [34].

More recently Hrach et al. (2020) [28] looked at transcriptome changes in *dys-1(eg33)* and *dys-1(cx18)*, specifically from the body muscles and at different stages of disease progression. The gene expression patterns from these two strains had distinct differences and *dys-1(eg33)* showed aberrant splicing events (*dys-1(cx18)* was not tested). In their presymptomatic group (embryo to L2), they identified enrichment in genes associated with locomotion and larval development which is consistent with the locomotion defects that are detected in later life. There was also an abundance of genes involved in mitochondrial function, implying mitochondrial dysfunction occurs in early disease. At later stages of disease, the most abundant group of genes identified were related to myofibrillar assembly [28].

## 4. Physical Protein Interactions with DYS-1

### 4.1. DGC Associated Proteins

The dystrophin protein plays a structural role as part of the DGC and as discussed earlier many of the components of the mammalian DGC have been identified in *C. elegans* (Figure 2). The nature of the interactions between DYS-1 and other relevant proteins will be discussed further in this section.

Following the initial finding of DYS-1 in 1998, a handful of other proteins have been found to interact directly or indirectly with it. DYS-1 was found to interact directly with dystrobrevin (DYB-1) and syntrophins (STN-1 and -2) using the GST fusion protein technique and coimmunoprecipitation, providing the first evidence that a DGC may exist in *C. elegans* [10,29,38,39]. Orthologues were also found for other DGC members including, δ/γ-sarcoglycan (SGN-1), α- and β-sarcoglycans (SGCA-1 and SGCB-1), and dystroglycan (DGN-1), although the nature of these interactions is currently unknown [10]. There is some controversy regarding DGN-1. Initially DGN-1 was thought to associate with DYS-1 based on its homology with its human counterpart [10]. However, a subsequent study showed that it was found in the neurones and not the muscle, and that it functioned independently of DYS-1, implying that the initial proposed DGC structure shown in Figure 2 was incorrect [40]. More recent transcriptome studies have repeatedly identified *dgn-1* in their *C. elegans* muscle transcriptome, suggesting that there could be small amount of DGN-1 in *C. elegans* muscle after all [28,41,42]. Other proteins found to interact directly with DYS-1 via yeast two hybrid assay include the G protein GPA-13 [43], the cytoskeletal protein CTN-1 [44] and the dense body protein DEB-1 (vinculin) [45,46].

Further proteins have been shown to have indirect relationships with DYS-1 by connecting with proteins that have direct associations with DYS-1. For example, STN-1 has been found to associate with DYB-1 as well as DYS-1, and also SNF-6 which encodes an acetylcholine/choline transporter [47]. STN-2 acts as a linker molecule to associate DYS-1 and SAX-7 (a homologue of the L1 family of cell adhesion molecules) to ensure the maintenance of neural integrity [39]. CTN-1 also associates with DYB-1, and this interaction is important for localisation at the dense body [44].

### 4.2. Dense Body Signalling Proteins

In vertebrates, dystrophin is localised at costameres (consisting of the focal adhesion complex, the DGC and a spectrin-based filament network), at the sarcolemma and at the neuromuscular junction (NMJ). Costameres link the sarcomeres to the sarcolemma through an interaction with Z-disks [3]. In *C. elegans* dense bodies are the functional equivalent of Z-disks and costameres. As previously mentioned, DYS-1 interacts with DEB-1 (vinculin), and is localised at the base of the dense bodies [46]. The ZYX-1 zyxin protein is localised at and interacts with DEB-1, as does ATN-1 (α-actinin). ZYX-1 and ATN-1 also have a direct interaction and it has been shown that the localisation of ZYX-1 at the dense bodies is ATN-1-dependent [45]. ZYX-1 also interacts with DYC-1 (a CAPON related protein) resulting in the localisation of DYC-1 to the dense bodies as well [48].

### 4.3. Calcium Homeostasis Associated Proteins

**Figure 3:**
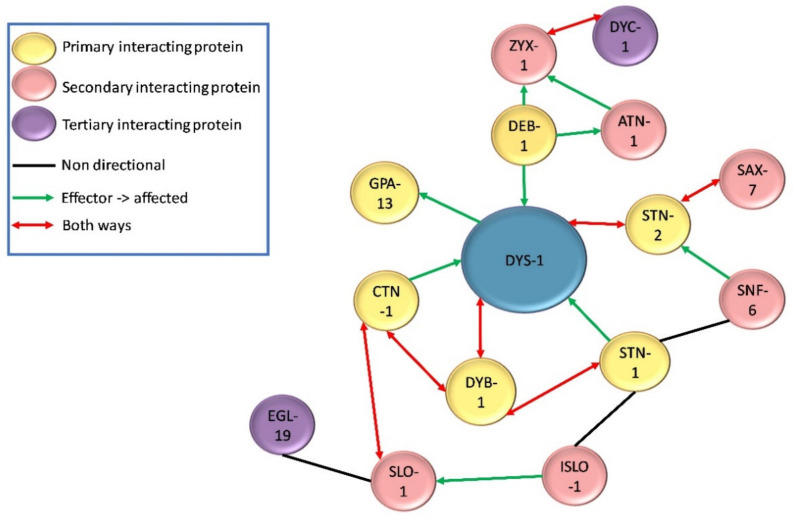
Nature of direct and indirect physical interactions with DYS-1.

**Figure 4:**
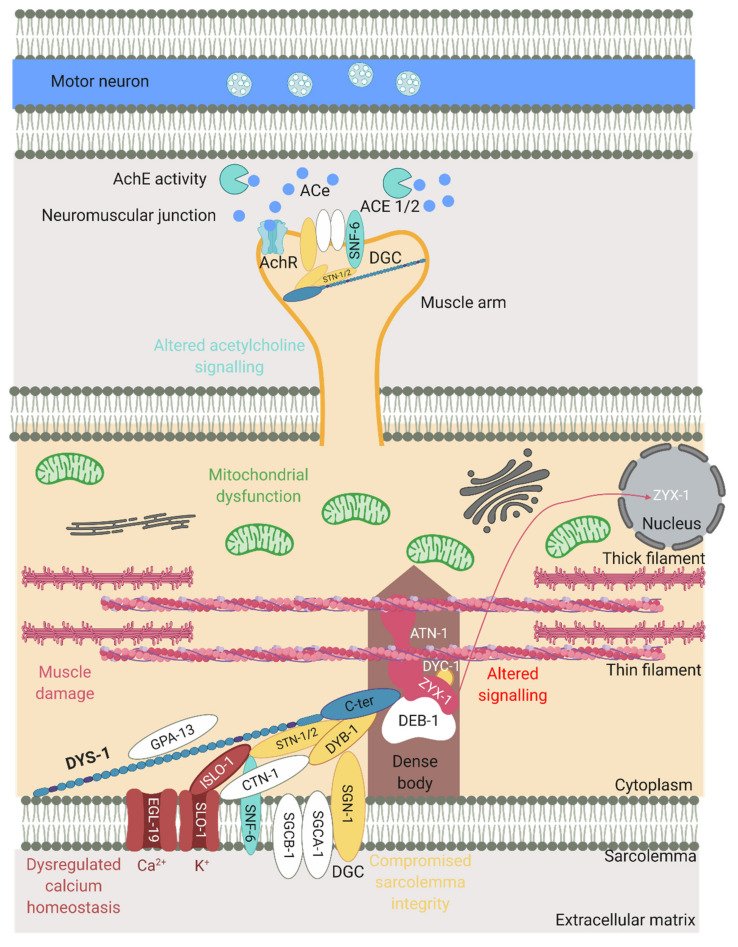
The effect of the loss of dystrophin on various processes in the mutants. Schematic of part of a muscle cell including a dense body anchored to the sarcolemma and actin/myosin filaments. The muscle arm is in close contact with a motor neuron and shows elements of the NMJ (acetylcholine (ACe), acetylcholinesterase (AchE), acetylcholine receptor (AchR)). DYS-1 has many key roles in muscle function as does the DGC that can be seen in the middle. Proteins in yellow represent those that are important for maintaining sarcolemma integrity, those in pink are muscle related, those in red are calcium related and those in blue are acetylcholine signalling related. Processes associated with loss of dystrophin can also be seen. Adapted from Gieseler et al. (2017) [15]. Created with biorender.com.

Another important group of proteins that have an indirect association with DYS-1 are those that have a role in controlling and maintaining calcium homeostasis. Dysregulation of calcium homeostasis is commonly seen in DMD models including *C. elegans* [49,50]. SLO-1 is a large conductance potassium channel activated by intracellular calcium and voltage fluctuations. It is present in neurones, where it helps to regulate neurotransmitter release, and in muscles where it is localised at both the M-line and the dense bodies, close to the L-type calcium channel EGL-19. SLO-1 has been found to be mislocalised in *dys-1* mutants, its localisation is thought also to depend on CTN-1 [51,52,53]. ISLO-1 is a DGC interacting gene, it acts as an adapter molecule and physically interacts with SLO-1 and STN-1, connecting it to the DGC. SLO-1 channels are stimulated by calcium entry and are often localised to calcium rich areas. The corresponding mediated potassium efflux is thought to attenuate calcium-dependent activation of muscle and prevent hyper-excitation and hyper-contraction of muscle in response to large calcium increases., thus involving DYS-1 and the DGC in the control of calcium homeostasis [52]. The nature of these interactions are displayed in Figure 3 and Figure 4.

## 5. Genetic Interactions with *dys-1*

The dystrophin gene was first identified in *C. elegans* using a forward genetics approach [20]. Humans have three dystrophin genes (dystrophin, utrophin, DRP2) whereas *C. elegans* only has one; *dys-1* was mapped to the right arm of chromosome I and is made of 46 exons spanning 13 kb. A list of phenotypes associated with these mutants can be found in Table 2. To test whether the *dys-1* gene was functionally similar to human dystrophin, *dys-1* mutants were given a transgene carrying the putative regulatory regions of the *dys-1* gene fused to the open reading frame of the human dystrophin gene and this partially rescued the hyperactivity phenotype of the mutant animals [20]. Over the following years, several genes were shown to participate in the same biological function as *dys-1*, had altered activity due to *dys-1* deficiency or could be modified to compensate for the absence of *dys-1* (Table 3 and Figure 4).

### 5.1. Dystrophin-Like Genes

A handful of genes when mutated in *C. elegans* give phenotypes such as *dys-1* mutants (hyperactivity, head bending and a tendency to hyper contract), suggesting they may participate in the same biological function as *dys-1*. These genes encode for proteins that are also members of the DGC or interact with the DGC, highlighting the importance of maintaining the integrity of the DGC. These include *dyb-1* (dystrobrevin) [54], *dyc-1* (capon) [24], *stn-1/2* (syntrophins) [55], *sgn-1* (sarcoglycan) [10], *snf-6* (an acetylcholine transporter) [47], *slo-1* (a potassium channel) [51] and *islo-1* (interactor of *slo-1*) [52] (Figure 2 and Figure 4). *dyb-1* and *sgn-1* like *dys-1* single mutants do not show muscle degeneration, but in an *hlh-1* sensitised background they do and to similar degrees as *dys-1(cx18);hlh-1(cc561)* [10,54,56]. Importantly, there is some evidence to suggest that overexpressing one of these genes in the *dys-1* mutants, can partially compensate for the absence of dystrophin. For example, overexpressing *dyb-1* in *dys-1(cx18);hlh-1(cc561)* mutants helps to delay the onset of incoordination and muscle cell damage and overexpressing *dyc-1* partially reduces locomotion and egg laying defects [24,57].

### 5.2. Muscle Related Genes

*C. elegans* body wall muscles resemble that of vertebrate skeletal muscle in that they are comprised of sarcomeres. Interestingly, introducing mutations in genes that have a role in muscle contraction in the *dys-1(cx18);hlh-1(cc561),* resulted in suppression of the muscle degeneration usually observed. These included *unc-22* (twitchin), *unc-89* (obscurin), *unc-96* (a M-line protein), *lev-11* (tropomyosin) and *pat-10* (troponin C) [58]. This suggests that reducing sarcomere contraction can slow down muscle degeneration as it reduces the physical tension on the muscle fibres. This is supportive of evidence in mouse models suggesting that denervation and immobilisation of skeletal muscle could be a beneficial treatment in patients [59,60,61]. In addition, triple mutants of *dys-1(cx18);hlh-1(cc561)* with *atn-1* (actin) or *zyx-1* (zyxin) also show a reduction in muscle degeneration. Surprisingly, overexpression of *zyx-1* in the double mutant also reduced muscle degeneration but to a lesser degree [45]. This implies that the ZYX-1 protein could be involved in the muscle degeneration process and targeting zyxin protein could be an effective treatment in DMD.

### 5.3. Calcium Related Genes

One of the pathophysiologic mechanisms in DMD patients is loss of calcium homeostasis resulting in an increase in intracellular calcium levels [62]. It has recently been suggested that calcium increases in the sarcoplasmic reticulum occurs before any other phenotype, which is detrimental to muscle health [50] and there is evidence to show the mislocalisation of some calcium channels in the absence of *dys-1,* which may be responsible for this rise [51,52,63].

Inhibition of some calcium channels significantly reduces muscle degeneration and thus with further study could be a beneficial treatment in DMD. Inhibiting *egl-19* (L-type calcium channel) [64], *unc-2* (voltage-sensitive calcium channel) [58], *unc-68* (ryanodine receptor) [65], and *itr-1* (inositol triphosphate receptor) [66] in the *dys-1(cx18);hlh-1(cc651)* model resulted in a reduction in muscle degeneration. This is likely due to reduced calcium entry into the cell and reduced release of calcium from the sarcoplasmic reticulum to the cytosol.

Sarcoplasmic calcium leakage is also a characteristic in DMD and is thought to mediate myofiber death [67]. It is therefore reasonable to propose that altering intracellular calcium movement could be a potential treatment as well. RNAi has been used to inhibit *cmd-1* (calmodulin), *sca-1* (SERCA) and *csq-1* (calsequestrin) in *dys-1(eg33)* animals. Of these, only a reduction in calmodulin was able to improve function in the dystrophic worms by returning calcium levels to those of WT and improving calcium clearance during contraction/relaxation cycles [50]. This result was unexpected as in the mouse model inhibition of calmodulin was found to be detrimental, but calmodulin levels have been found to be elevated in human dystrophic muscle; therefore, targeting calmodulin could be a potential avenue but further work is required to fully understand calmodulins role in DMD [68,69].

Finally, calpains, which are calcium activated regulated protesases, have been implicated in muscular dystrophy, and are known to be activated by increased intracellular calcium. Inhibition of calpains has given a histological improvement in the *mdx* mouse model [70]. There are two classes of calpains, typical or atypical, but *C. elegans* only express atypical calpains, and one of these genes is *clp-1*. *dys-1(cx18);hlh-1(cc561ts);clp-1(tm690)* mutants had almost half the number of degenerated muscle cells compared to *dys-1(cx18);hlh-1(cc561ts)* mutants, providing another target for treatment [71].

### 5.4. Excitation–Contraction Coupling Genes

There is some evidence to suggest that improving the excitation–contraction coupling defect seen in DMD could be beneficial but requires further investigation. Excitation–contraction coupling is initiated by the release of acetylcholine from the axon terminal and the binding of acetylcholine to receptors on the sarcolemma, triggering a muscle action potential. The acetylcholine is then removed from the synaptic cleft by acetylcholinesterase. One of the early phenotypes identified in the dystrophin mutants was a hypersensitivity to acetylcholine and aldicarb (altered cholinergic signalling), and a decline in acetylcholinesterase activity [8,20]. In *C. elegans,* the genes that exhibit acetylcholinesterase activity are *ace-1* and *ace-2*. An interaction has been identified between *dys-1* and the *ace* genes as *dys-1* mutants show lower acetylcholinesterase activity, but the nature of this interaction is unknown [8]. *snf-6* (which is responsible for clearing acetylcholine from the cholinergic synapse) becomes mislocalised when dystrophin is absent, resulting in an increase in acetylcholine at the NMJ and insufficient acetylcholinesterase to break it down. Therefore, cholinergic transmission is likely upregulated in the absence of dystrophin [72]. Mariol et al. have investigated the excitation–contraction cascade by using RNAi to knock down genes linked to neurotransmitter release, acetylcholine signalling and others. Inhibition of *unc-13* (gene required for neurotransmitter release at the NMJ), *unc-38* and *unc-29* (both genes that encode for the acetylcholine receptor), in the *dys-1(cx43);hlh-1(cc561)* model led to a strong reduction in muscle degeneration [58].

The muscle action potential results in the release of calcium from the sarcoplasmic reticulum, which binds to troponin, displacing tropomyosin and allowing cross bridges to form between actin and myosin. Several genes mentioned in the previous section on calcium also play a role in this process either through calcium influx or calcium removal. L-type calcium channels such as *egl-19* are triggered by the action potential to release calcium into the cell and this influx can cause further release of calcium from the sarcoplasmic reticulum through ryanodine receptors (*unc-68*). This increase in calcium initiates contraction, and relaxation occurs through the clearance of calcium through channels such as SERCA (*sca-1*). As discussed previously, inhibition of calcium release channels significantly reduced muscle degeneration in this model but inhibition of clearance channels did not [50,64,65]. This implies that reducing calcium levels is likely to be important in the treatment of DMD.

### 5.5. Mitochondrial Genes

Mitochondrial dysfunction is a further underlying mechanism associated with DMD. Mitochondrial permeability transition pores (mPTP) are multiprotein complexes found in the inner mitochondrial membrane under certain pathological conditions [73]. An example is high concentrations of intracellular calcium, as in DMD, which can cause the opening of the mPTP through cyclophilin D and the release of cytochrome c which may trigger loss of functional mitochondria, reduction in ATP production and apoptosis/cell death. RNAi knock down of *cyn-1* (a cyclophilin D orthologue), and *cyc-2.1* (a cytochrome c orthologue), led to a significant reduction in muscle degeneration and an improvement in locomotion in *dys-1(cx18);hlh-1(cc561)* mutants [66]. The question regarding the nature of the relationship between the mitochondria and calcium transport in *C. elegans* remains open—for example, do ANT2, MCU, and NCLX play similar roles in DMD pathology in worms as they do in higher animal models [74,75,76].

It has also recently been found that dystrophin-dependent muscle degeneration is quickly followed by an increase in mitochondria fragmentation and apoptosis. Decreasing mitochondrial fission by inhibiting *drp-1* (mitochondrial fission gene) or *ced-3* (cleaves DRP-1) or increasing mitochondrial fusion by overexpressing *eat-3* or *fzo-1* (inner and outer mitochondrial membrane fusion, respectively) in the *dys-1(cx18);hlh-1(cc561)* model results in a reduction in mitochondrial fragmentation and fewer abnormal muscle cells [27]. In addition to altering mitochondrial dynamics, inhibiting genes involved in apoptosis and DNA degradation could be a potential treatment for DMD. The inhibition of *wah-1* (orthologue of human AIF), *cps-6* (orthologue of human EndoG), *crn-2* (orthologue of human TATDN1), *ced-1* (cell-corpse recognition), and *psr-1* (migration of engulfing cells) in *dys-1(cx18);hlh-1(cc561)* also caused a decline in the number of abnormal muscle cells, suggesting that they all participate in cell death upon dystrophin-muscle degeneration [27].

### 5.6. Other Signalling Genes

Dystrophin has a variety of signalling influences including calcium, acetylcholine and mitochondrial. Dystrophin has also been shown to positively modulate the EGF-Ras-MAPK pathway, which is important during vulval development in *C. elegans*. Inactivation of *dys-1* strongly suppressed the induction of ectopic vulvae by an activated *let-60* (*Ras* gene), highlighting dystrophins role in regulating EGF signalling during vulva induction [77]. Dystrophin knock down has been shown to lead to a disruption in protein homeostasis, which consequently leads to an increase in cellular stress levels in the muscle. One gene that has been shown to become overexpressed in *dys-1(eg33)*, is *gst-4*, a transcriptional reporter induced by oxidative stresses. One pathway that is known to enhance stress resistance is insulin growth factor (IGF) signalling. *daf-16*, a FOXO gene, and *daf-2*, the gene encoding for an IGF receptor, can be targeted to reduce IGF signalling. *dys-1(cx18/eg33);daf-2(e1370)* mutants are able to protect the muscle from cell death and increase lifespan. This improvement relies on *daf-16*, as in *daf-2(e1370);daf-16(mu86);dys-1(cx18)* triple mutants, this positive effect was ameliorated [13]. Manipulation of these signalling pathways could also be a target for future studies in DMD.

Finally, there are a handful of other genes that have been shown to have a genetic interaction with *dys-1* but not much is known about the nature of these interactions. For example, knock down of *hlh-1* (MyoD ortholog) has been used to give muscle degeneration in the *dys-1* single mutants but mechanisms are unclear [24]. Downregulating *chn-1* (CHIP family) suppresses muscle degeneration in the *dys-1(cx18);hlh-1(cc561)* model, likely by reducing proteasome activity [78]. RNAi knock down of *cah-4* (carbonic anhydrase) [79] and *gdi-1* (rab guanine-nucleotide dissociation inhibitor) [80] in the double mutant also reduced muscle degeneration, but again the mechanisms remain unclear.

## 6. Pharmacological Interventions Trialled in *dys-1* Mutants

**Figure 5:**
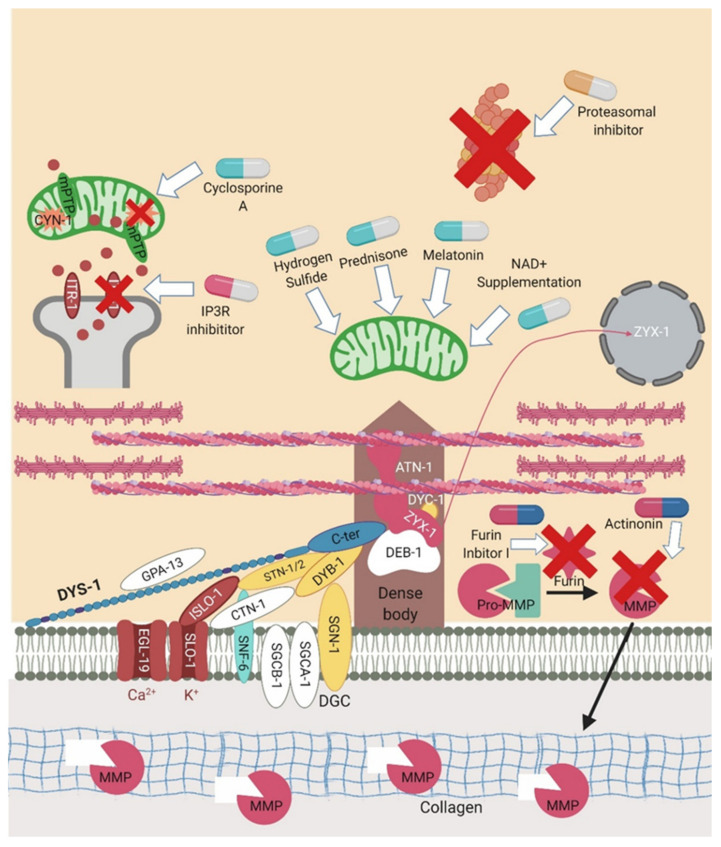
Sites of action for pharmacological interventions. *C. elegans* has proven itself as a good drug screening platform for DMD. A number of these interventions are represented in the above figure. The pills in light blue and white represent the majority of the trialled drugs that are acting to improve mitochondrial dysfunction. Those in pink and white are altering calcium signalling. The orange and white pills are proteasomal inhibitors which act to reduce proteasome activity. Those in pink and blue represent extracellular matrix targeting compounds that aim to reduce the breakdown of collagen. Created with biorender.com.

*C. elegans* has not only proven itself as a useful model for studying human diseases but also as a tool for drug discovery [81]. Several pharmacological interventions have been rationalised and tested in the *dys-1* mutants with varying success. The translatability of some of these interventions from the worm model remains unclear. The different treatments that have been trialled can be found in Table 4 and their proposed sites of action can be seen in Figure 5.

### 6.1. Glucocorticoids

The standard pharmacological treatment given to DMD patients are glucocorticoids, of which prednisone is the most common. In a blind drug screen of ~100 compounds in the *dys-1* double mutant, prednisone was identified as the most successful in reducing the number of abnormal muscle cells, highlighting the utility of this model in drug screening [82]. There are several hypotheses on the mechanisms of action of prednisone, yet it is not entirely clear. It is proposed that prednisone may decrease inflammation, but as *C. elegans* largely lack a conventional inflammatory system, it is probable that it is having other effects as well. In a recent article, prednisone was shown to improve movement and strength in the *dys-1(eg33)* model through improvements in the structure of the mitochondrial network and oxygen consumption [11,26]. Deflazacort is another glucocorticoid which has now received US FDA approval for the treatment of DMD; despite exploration of other models [85], studies have yet to commence in *C. elegans*.

### 6.2. Hormone Related Therapies

Hormone related drugs have also been shown to reduce muscle degeneration and improve muscle function in the *dys-1* model [11,26]. Interventions that modified serotonin levels were shown to be beneficial in *C. elegans,* including serotonin itself, drugs that inhibit serotonin reuptake (Fluoxetine (Prozac), Imipramine and Trimipramine) and serotonin agonists (m-chlorophenyl piperazine and N-methyl quipazine) [83]. Serotonin treatment has also been shown to be beneficial in zebrafish *sapje* model of DMD and in the *mdx* mouse model [86,87]. Mechanistic insight is lacking; there could be reduced serotonin levels in the absence of dystrophin, so the interventions are simply replacing what is lacking. However, in a small cohort of human patients, serotonin levels were found to be comparable to healthy controls in plasma but lower than controls in platelets [88,89]. Melatonin is another hormone that has been shown to improve muscle function in *C. elegans*, *mdx* mice and in patients. This likely acts by reducing oxidative stress [11,90, 91, 92].

### 6.3. Proteasome Inhibitors

Proteasome degradation has been shown to decrease dystrophin levels in DMD patients and so providing treatments that focus on stabilising the mutant protein could be beneficial [93]. Reducing proteasome activity by downregulating *chn-1* has been shown to reduce muscle degeneration in the *dys-1* model [78]. As gene knockout is currently not a feasible treatment in humans, MG132 was trialled as well which is a nonspecific proteasome inhibitor. When administered at low doses it can inactivate *chn-1* and thus block proteasome degradation in the *dys-1* model [78]. MG132 treatment has also been trialled in the *mdx* mouse model and in freshly isolated skeletal muscle biopsies, in both of which the inhibitor rescued the expression of the DGC (likely due to rescuing the cell membrane localisation) [94,95, 96]. However, it seems unlikely that proteasome inhibitors as a stand-alone treatment will be able to provide a cure for DMD, but they may offer potential as part of a “therapeutic cocktail” [97].

### 6.4. Sulphonamides

In a *C. elegans* DMD model screen of 1000 currently approved treatments, carbonic anhydrase inhibitors were identified among the top hits [79]. Previously, carbonic anhydrase levels in plasma were shown to be elevated and carbonic anhydrase inhibitors have had some success in human pilot studies [98,99, 100]. In this screen, the two compounds identified were the sulphonamides methazolamide and dichlorphenamide, which are thought to act by inhibiting *cah-4*. As previously mentioned, RNAi against *cah-4* was also beneficial in the *dys-1 C. elegans* model. These drugs were trialled in both *C. elegans* and in the *mdx* mouse with a decline in muscle degeneration seen in *C. elegans* and an increase in force in the mouse model [79]. It is proposed that these compounds act by modifying the pH, which alters the transmembrane potential and excitability.

### 6.5. Compounds Targeting the Mitochondria

Recently, the mitochondria have become an attractive target for drug treatments in DMD. A chemical screening tool was developed for the study of neuromuscular disorders in *C. elegans* [101]. Using these methods, two therapies were identified that targeted the mitochondria. The first was low dose cyclosporine A, thought to act by inhibiting cyclophilin D, *cyn-1*. It is hypothesised that this drug reduced muscle degeneration through the regulation of mPTP opening [66]. This has also been reported in the *mdx* mouse model, but a clinical trial in patients did not show significant improvements in muscle function [102,103]. Alisporvir is another cyclophilin inhibitor which has been shown to restore maximal respiratory capacity in DMD patient cells so could potentially be a better candidate to trial [104]. The second compound identified was the IP3R inhibitor aminoethoxydiphenyl borate. This inhibits the calcium channel ITR-1 which has been shown to have a role in dystrophin-dependent muscle degeneration [66]. It has been shown recently in the *mdx* mouse model that the IP34 receptor has a role in increasing basal cytoplasmic calcium and blocking this receptor restores muscle function [105]. Another potential mitochondrial drug is NAD+ supplementation; NAD+ depletion has been shown to occur in patients with DMD; therefore, supplementation could be a beneficial treatment. NAD+ is also naturally occurring in the body so the risk of adverse side effects is low. NAD+ supplementation in DMD *C. elegans*, *mdx* mice and human cells has been shown to be beneficial [84]. The use of hydrogen sulphide (H_2_S) compounds has recently been suggested as another potential treatment for DMD. Supplementing *dys-1(eg33)* animals with the slow release H_2_S donor, sodium GYY4137 or the mitochondria targeted H_2_S donor AP39, improved movement and strength by correcting mitochondrial dysfunction [12].

### 6.6. Extracellular Matrix Targeted Compounds

Finally, targeting the ECM could also prove to be beneficial. Collagen has been shown to play a role in protecting muscle cells against dystrophy but is rapidly degraded by matrix metalloproteinases (which are activated by furin). Using a furin inhibitor (furin inhibitor I) and an inhibitor of matrix metalloproteinases (actinonin), proved beneficial in the double mutant as there was less degradation of collagen [65].

## 7. Conclusions

*C. elegans* has proven itself as a very useful model for studying DMD, especially more recently with the use of the *dys-1(eg33)* model and the development of more clinically relevant and translational assays. Since the mechanism of action of prednisone is largely unknown, improving mechanistic understanding of this drug could potentially identify targets that, at the moment, are not being addressed. One of the main benefits of this model continues to be in its ability to run high-throughput screens of compounds. This could lead to the discovery of novel treatments that could be used instead of prednisone without the side effects. Identifying a drug that could extend lifespan or health span in DMD patients would be a huge breakthrough in this field.

## Figures and Tables

**Table 1:** Comparison of the most commonly used animal models in the study of DMD.

Model Type	Benefits	Similarities to DMD in Humans	Limitations
*C. elegans*	Easy and cheap to maintain, short lifespan, high throughput experiments possible. Similar muscle structure and has orthologues for most human DGC proteins [10].	Display movement and strength decline [11], altered gait [12] and shortened lifespan [13].	Have a very simple body plan and nonconventional circulatory system [14]. Are unable to regenerate muscle as they lack satellite cells and do not have a conventional inflammatory system [15].
Zebrafish	Easy to house and care for, high throughput experiments possible. High skeletal muscle content and expresses orthologues of most human DGC proteins [16].	Changes in gait and lower activity [17].	Missing several mammalian organs, are ectothermic and are influenced heavily by their environment.
*Mdx* mouse	One of the easier mammalian models to house and care for with a relatively short lifespan. High genetic similarity to humans including a DGC [4].	Genetic and biochemical homologue of disease in humans. Displays ECG abnormalities and cardiomyopathy [18].	Minimal clinical symptoms (no loss of ambulation and muscle weakness is not displayed until ~15 months) and lifespan is not majorly reduced [19].
Dystrophin deficient rats	A convenient size as they are larger than mice allowing for studies with high statistical power but still relatively easy to house and care for. High genetic similarity including a DGC [5].	Muscles showed severe fibrosis, muscle weakness and reduced activity [5,6].	Not a well-established model and characterisation is still ongoing.
Golden retriever	Higher genetic similarity to humans compared to other mammalian models. Case reports showing that DMD occurs naturally in these animals as well.	Extensive homology in pathogenesis. Pathogenesis manifests in utero and extensive muscle necrosis can be seen and is progressive. They also have a shortened life span frequently dying from cardiac and respiratory failure [7].	Expensive to maintain, not easily genetically manipulable and many ethical concerns.

**Table 2:** Known phenotypes associated with the most common *dys-1* models.

Class of Phenotype	*dys-1(cx18)*	*dys-1(cx18;hlh-1)*	*dys-1(eg33)*
**Locomotion**	Exaggerated body bends, hyperactive, hypercontracted, overbent, swimming defective and burrowing defective [11,20,21,22,23].	Exaggerated body bends, hyperactive, hypercontracted, overbent and swimming defective [24,25].	Exaggerated body bends, hyperactive, hypercontracted, overbent, swimming defective and burrowing defective [11,12,22,23,26].
**Muscle structure**	Very little muscle degeneration [20].	Severe muscle degeneration [24].	Severe muscle degeneration [13].
**Response to neuromuscular agents**	Aldicarb hypersensitive, levamisole resistant [11,20].	ND	Levamisole resistant [11].
**Mitochondria structure and function**	Minor fragmentation of the mitochondria network, moderate depolarisation of mitochondrial membrane, no change in basal oxygen consumption rate [11].	Severe fragmentation of the mitochondria network [27].	Severe fragmentation of the mitochondria network, severe depolarisation of the mitochondrial membrane, elevated basal oxygen consumption rate [11,12].
**Life span**	Shortened life span [13,28].	ND	Shortened life span [12,13,28].
**Egg laying**	No defect noted [20].	Egg laying defect [24].	ND
**Strength**	Not detectably weaker than WT [11].	ND	Significant weakness detected compared to WT [11,12].

**Table 3:** Genes known to have a genetic interaction with *dys-1.*

Gene Classification	Associated Genes
**Dystrophin-Like**	*dyb-1, dyc-1, islo-1, snf-6, slo-1, sgn-1, stn-1/2*
**Muscle related**	*atn-1, lev-11, pat-10, unc-22, unc-89, unc-96, zyx-1*
**Calcium**	*clp-1, cmd-1, csq-1, egl-19, islo-1, itr-1, sca-1, slo-1, stn-1, unc-2, unc-36, unc-68*
**Excitation–contraction coupling**	*ace-1, ace-2, snf-6, unc-13, unc-29, unc-38*
**Mitochondria**	*ced-1, ced-3, cps-6, crn-2, cyc-2.1, cyn-1, drp-1, eat-3, fzo-1, itr-1, psr-1, wah-1*
**Other signalling**	*daf-2, daf-16, gst-4, let-60*
**Other**	*cah-4; chn-1, gdi-1, hlh-1*

Colours used in table correspond with sites of action displayed in Figure 4 and Figure 5.

**Table 4:** Pharmacological interventions tested in *dys-1* mutants.

Drug Class	Tested Models	Proposed Mechanism of Action
Glucocorticoids (Prednisone)	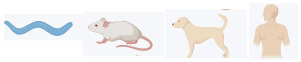	Unknown hypothesised to have a direct effect on striated muscles (likely by repairing dysfunctional mitochondria and the mitochondrial network) [11,26,82].
Serotonin	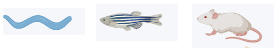	Unknown- as lack of *dys-1* is known to disrupt signalling pathways it could affect serotonin receptors and by replacing the serotonin you can reduce muscle degeneration [11,26,83].
Proteasomal inhibitor (MG132)	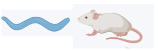	Inhibition of the proteasome rescues the protein localisation of the members of the DGC [78].
Sulphonamides (methazolamide and dichlorphenamide)	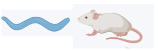	Inhibits *cah-4* [79].
Cyclosporine A	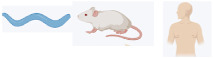	Inhibits *cyn-1* which blocks or delays mPTP opening [66].
IP3R inhibitor aminoethoxydiphenyl borate	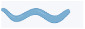	Inhibits *itr-1* [66].
Nicotinamide riboside supplementation	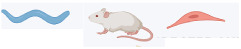	Increases NAD+ levels [84].
Melatonin	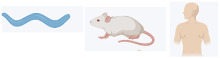	Reduces oxidative stress [11].
Furin inhibitor I	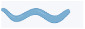	Inhibits Furin [65].
Actinonin	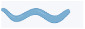	Inhibits matrix metalloproteinases [65].
Hydrogen sulphide	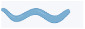	Improve mitochondrial dysfunction [12].

Colours used in table correspond with sites of action displayed in Figure 4 and Figure 5.
